# Supplementary material for: Recovery of Potential Starter Cultures and Probiotics from Fermented Sorghum (Ting) Slurries
Source: Microorganisms. 2023 Mar 9;11(3):715. doi: 10.3390/microorganisms11030715 (PMC10054160; doi:10.3390/microorganisms11030715)
Supplement: Supplementary file 1 [file microorganisms-11-00715-s001.zip › microorganisms-2177454-supplementary.pdf]

Table S1. (**a**). Molecular identification of 60 LAB isolates based on the 16S rRNA. (**b**). Comparison of 16S rRNA and *phes* gene sequencing ID of the 9 selected strains.

(a)

**Sample ID**	Identification	% composition of each strain
**D**	*Lactobalicillus helveticus*	60
	Lactobacillus amylolyticus	40
**K**	*Lacticaseibacillus paracasei subsp. paracasei*	52.9
	Lacticaseibacillus paracasei	47.1
**I**	*Lacticaseibacillus paracasei subsp. paracasei*	52.9
	Lacticaseibacillus paracasei	41.2
	Lactobacillus helveticus	11.8
**J**	*Lactiplantibacillus plantarum*	66.6
	Lacticaseibacillus paracasei	11.1
	Loigolactobacillus coryniformis	11.1
	Loigolactobacillus coryniformis subsp. *torquens*	11.1
**T**	*Lactiplantibacillusb plantarum*	57.1
	Levilactobacillus brevis	42.9

(b)

**Isolate code**	**16SrRNA**	**% ID**	***phes* Gene**	**% ID**
D12	*Lactobacillus amylolyticus*	99	*Lacticaseibacillus paracasei subsp. paracasei*	98
D7	*Lactobacillus helveticus*	99	*Lactobacillus helveticus*	97
T12	*Lactiplantibacillus plantarum*	99	*Lactiplantibacillus plantarum*	99
K5	*Lacticaseibacillus paracasei*	99	*Lacticaseibacillus paracasei*	99
K20	*Lacticaseibacillus paracasei*	99	*Lacticaseibacillus paracasei*	98
I12	*Lacticaseibacillus paracasei subsp. paracasei*	99	*Lacticaseibacillus paracasei subsp. paracasei*	98
J20	*Loigolactobacillus coryniformis subsp. torquens*	99	*Loigolactobacillus coryniformis subsp. coryniformis*	98
J22	*Loigolactobacillus coryniformis*	99	*Loigolactobacillus coryniformis*	96
T8	*Levilactobacillus brevis*	99	*Levilactobacillus brevis*	99

Table S2. Antibiotic profile of the LAB strains against selected antibiotics.

Bacterial strains	AMP	ER	MUP	TET	C	PB	STEP	K	OX
K5	+++	+++	++	+++	++	-	-	-	-
K20	+++	+++	++	+++	++	-	-	-	-
J20	+++	+++	+	+++	+++	++	+	-	-
J22	+++	+++	++	+++	+++	++	-	-	-
T12	+++	+++	++	++	+++	-	-	-	-
T8	+++	+++	++	++	+++	++	+	-	-
D12	+++	+++	++	+++	++	-	-	-	-
D7	+++	+++	++	++	++	-	-	-	-
I12	+++	+++	++	+++	++	-	-	-	-
V	+++	+++	++	++	+++	-	-	-	-
Y	++	++	++	++	+++	-	-	-	-

(-) no inhibition, (+) zone of inhibition between 5 and 10 mm, (++) zone of inhibition between 10 and 20,. (+++) zone of inhibition > 20 AMP, ampicillin; Erythromycin; MUP, mupirocin; TET, tetracycline; C, chloramphenicol; STREP, streptomycin; K, kanamycin; OX, oxacillin.

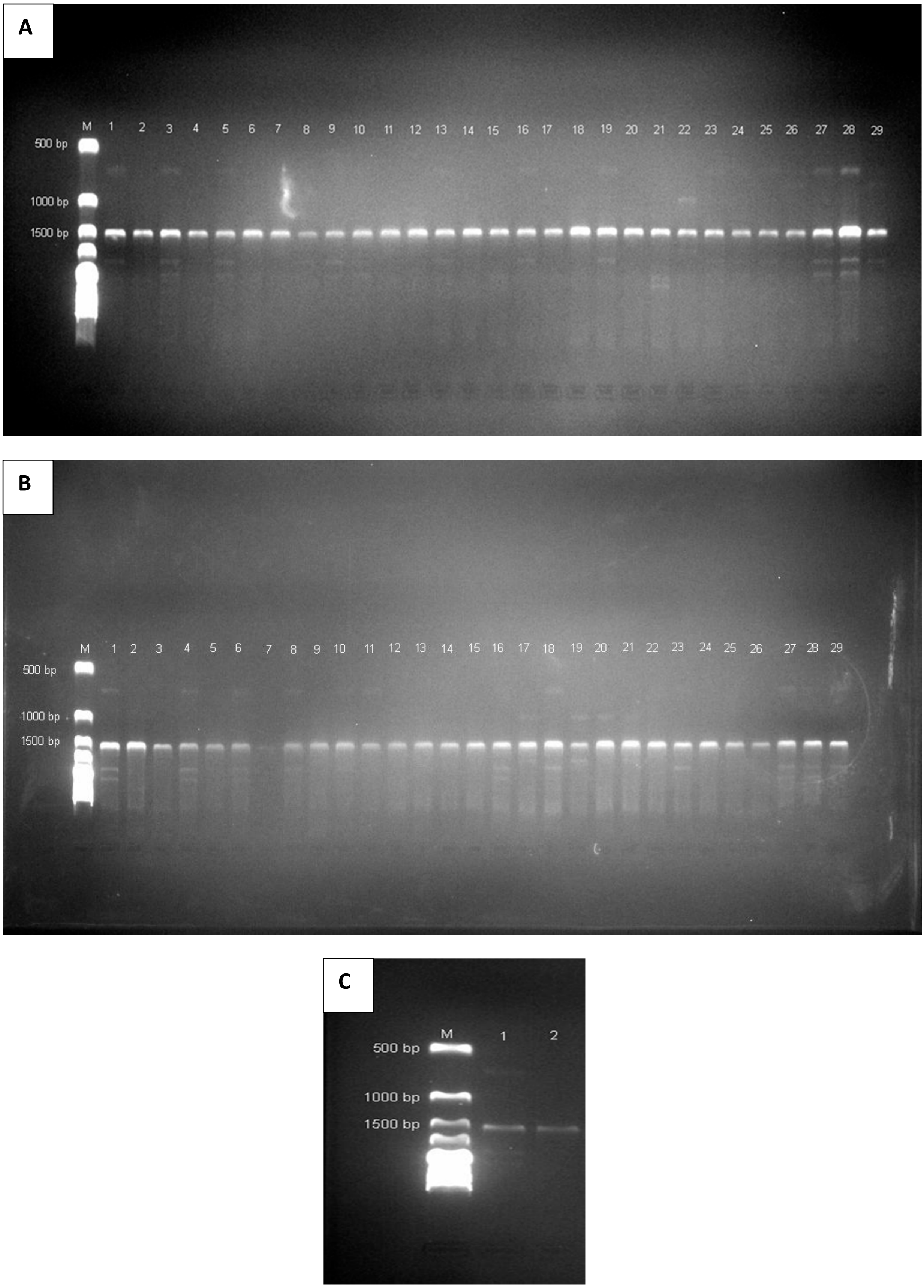

Figure S1: Electrophoresis analysis of amplified 16S rRNA gene of isolates. All the isolates yielded DNA fragments of 1466bp. Lane M is a 1kbp ladder, line 1 to 29 amplified DNA fragments (A-B). Lane M is a 1kbp ladder, well 1 to 2 amplified DNA fragments (C).

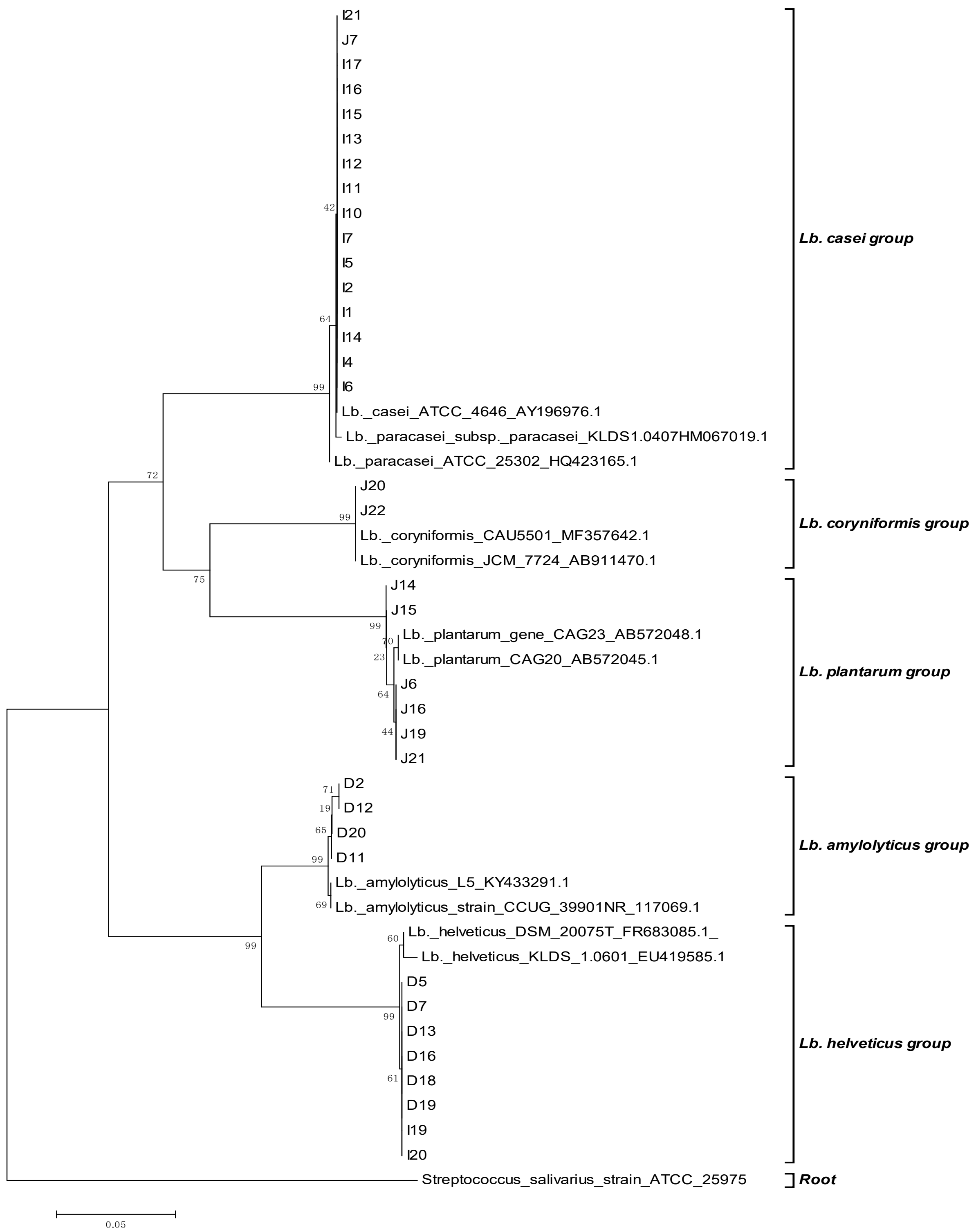

Figure S2a: The phylogenetic tree illustrating the evolutionary relationships between study isolates and reference strains. The tree was constructed using the 16S rRNA sequences and neighbour-joining-method (Tumara & Nei, 1993). Bootstraps of more than 60% are indicated at the internodes. The reference strains from the NCBI data base are indicated with accession numbers. The phylogenetic tree was rooted by *S. salivarius* ATCC 25975 FJ154797.1. Bar 0.05 substitutions per site.

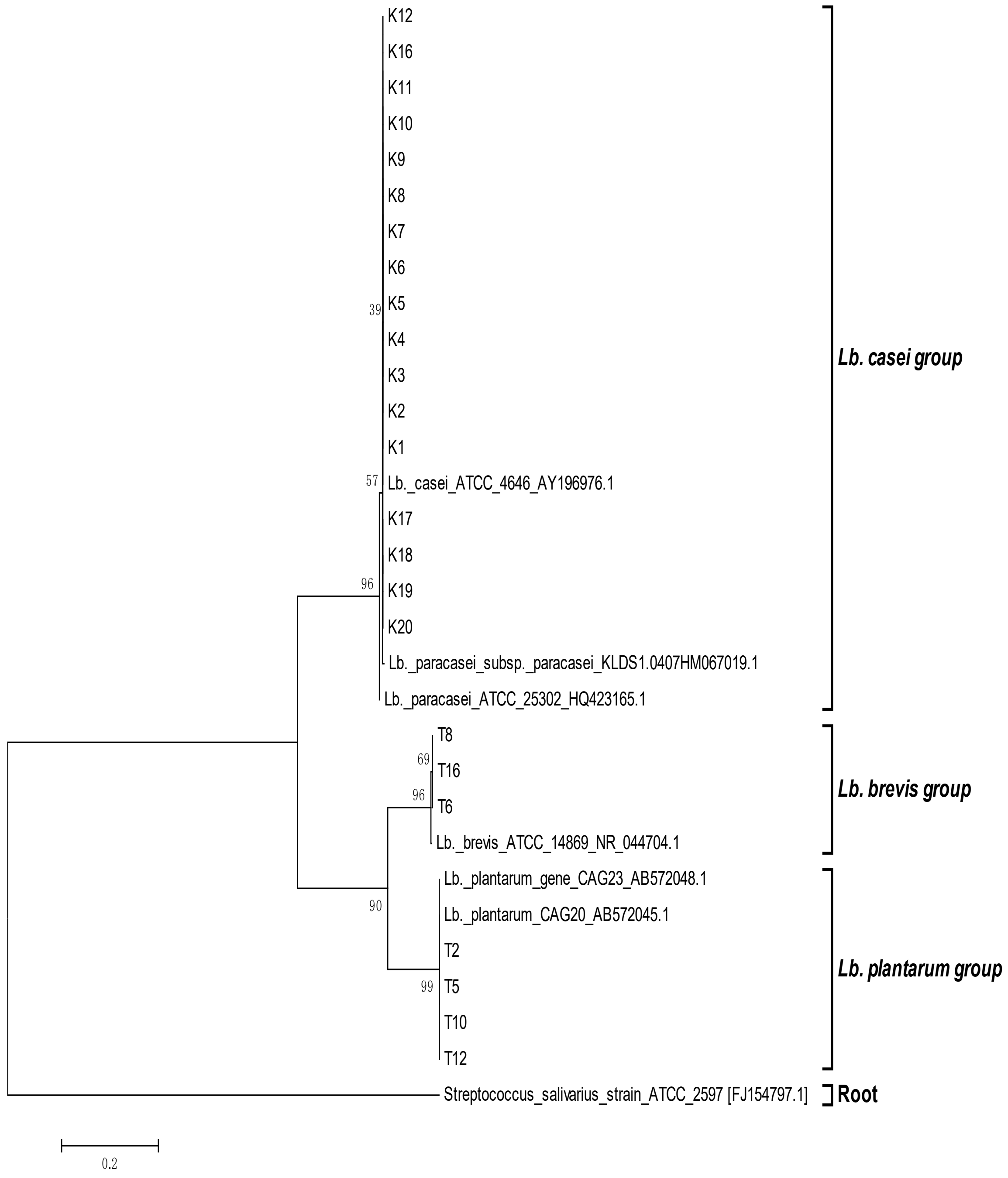

Figure S2b: The phylogenetic tree illustrating the evolutionary relationships between study isolates and reference strains. The tree was constructed using the 16S rRNA sequences and neighbour-joining-method (Tumara & Nei, 1993). Bootstraps of more than 60% are indicated at the internodes. The reference strains from the NCBI data base are indicated with accession numbers. The phylogenetic tree was rooted by *S. salivarius* ATCC 25975 FJ154797.1

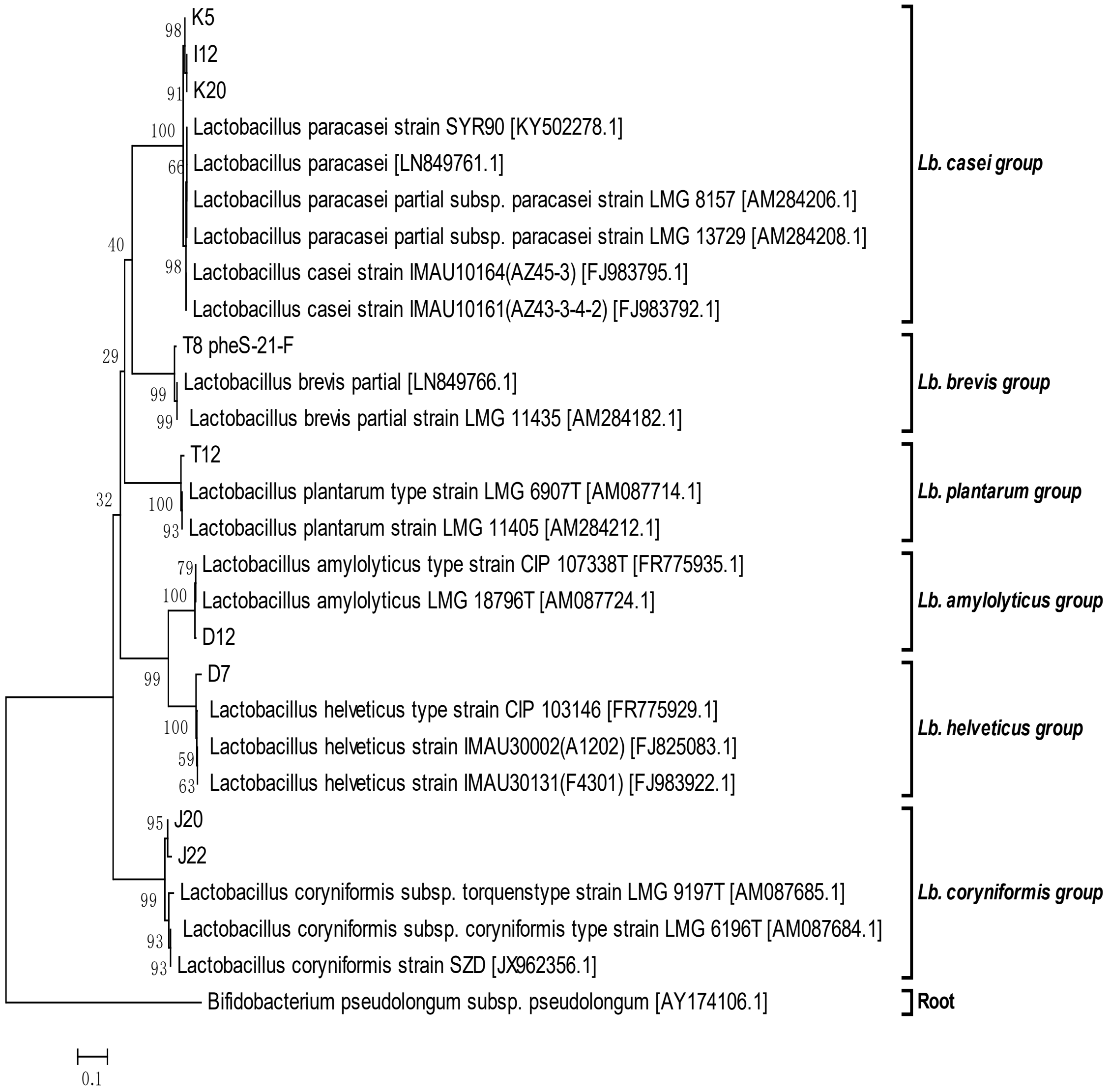

Figure S2c: The phylogenetic tree illustrating the evolutionary relationships between study isolates and reference strains. The tree was constructed using the *pheS* gene sequences and neighbour-joining-method (Tumara & Nei, 1993). Bootstraps of more than 60% are indicated at the internodes. The reference strains from the NCBI data base are indicated with accession numbers. The phylogenetic tree was rooted by *Bifidoacterium pseudolongum* subsp. *pseudolongum* AY174106.1. Bar 0.05 substitutions per site

Table S3. Viability of LAB strains incubated in phosphate buffered saline at pH 3 and 5 for 24 hours at 37 °C.

Strains	pH3					pH5				
	0 Hour	1 Hour	2 Hours	3 Hours	24 hours	0 Hour	1 Hour	2 Hours	3 Hours	24 hours
K5	7,23±0,07	6,665±0,26	6,725±0,60	6,61±0,71	6,395±0,05	7,3±0,03	6,74±0,37	7,1±0,28	7,59±0,03	6,57±0,33
K20	7,095±0,08	7,355±0,18	7,345±0,09	7,165±0,30	6,89±0,16	8,08±0,05	7,365±0,085	7,56±0	7,535±0,025	7,19±0,01
J20	7,5±0,03	6,87±0,04	7,245±0,02	7,52±0,06	6,33±0,01	7,29±0,04	7,33±0,07	7,31±0,01	7,605±0,11	6,35±0,04
J22	7,4±0,08	7,5±0,13	7,475±0,02	7,6±0,10	6,655±0,02	7,395±0,05	7,555±0,11	8,155±0,06	7,57±0,07	6,33±0,18
T12	8,33±0,01	8,835±0,03	8,07±0,04	7,995±0,06	7,085±0,05	7,875±0,60	7,11±0,09	7,095±0,02	7,4±0,11	6,99±0,01
T8	7,69±0,13	7,69±0,13	7,54±0,08	7,45±0,21	6,465±0,02	7,54±0,03	7,735±0,06	7,735±0,06	7,97±0,10	6,345±0,16
D12	4,3±0.06	0	0	0	0	0	0	0	0	0
D7	6,24±0,34	4,24±0,34	0	0	0	7,3±0	6,15±0,21	5,54±0,08	6,3±0	5,365±0,09
I12	7,22±0.06	7,475±0,19	7,79±0,07	7,605±0,04	6,635±0.04	7,22±0.06	7,475±0.06	7,46±0.04	7,58±0.03	6,53±0.20
V	6,69±0,01	6,59±0,09	6,36±0,11	6,38±0,10	6,525±0,23	6,72±0.23	6,58±0.15	6,45±0.12	6,41±0.04	6,58±0.13
Y	7,7±0,08	7,635±0,02	7,655±0,01	7,435±0,08	7,635±0,01	7,65±0,16	7,64±0,01	7,665±0,08	7,65±0,03	6,625±0,02

Table S4: Health benefits of potential probiotic species.

Isolate code	Species name	Health benefits	Reference
D12	*Lactobacillus amylolyticus*	*Promotes the hydrolysis of protein into amino acid, improving the nutritional quality and digestibility of cereal grains*	[1]
D7	*Lactobacillus helveticus*	*Indirectly benefit the human host by enhancing the bioavailability of nutrients, removing allergens and other undesired molecules from food, and producing bioactive peptides through the digestion of food proteins*	[2]
T12	*Lactiplantibacillus plantarum*	*Regulate the intestinal microbiota composition in a good way*	[3]
K5	*Lacticaseibacillus paracasei*	*Antimicrobial and antibiofilm activity; immune system stimulation; anti-inflammatory, antioxidant, anti-obesity and anti-proliferative/proapoptotic, lipid metabolism improving, hypocholesterolemic and stress modulator effects; and the enhancement of intestinal bacterial microbiota; among others.*	[4]
K20	*Lacticaseibacillus paracasei*	*Protection against pathogens, immunomodulation and anti-inflammatory, prebiotic, antioxidant and antiproliferative activity*	[4]
I12	*Lacticaseibacillus paracasei subsp. paracasei*	*The bacteria colonise the intestinal tract first and then reinforce the host defence systems by inducing a generalised mucosal immune response, balanced T-helper cell response, self-limited inflammatory response and secretion of polymeric IgA*	[5]
J20	*Loigolactobacillus coryniformis subsp. torquens*	*Increase HDL-C and adiponectin levels, inhibition of fat accumulation in the liver, and*	[6]

		suppression of the expression of adipogenic and lipogenic genes (PPAR-γ and FAS) and TNF-α in adipose tissues.	
J22	*Loigolactobacillus coryniformis*	*Enhance the immune response*	[7]
T8	*Levilactobacillus brevis*	*Reduce inflammation related to intestinal bowel disease, potentially overcoming issues related to the preservation of probiotic viability.*	[8]
